# Pitfalls with the "chest compression-only" approach: the challenge of an unusual cause

**DOI:** 10.1186/1757-7241-18-45

**Published:** 2010-08-13

**Authors:** Bjørn Ole Reid, Eirik Skogvoll

**Affiliations:** 1 Dept. of Anaesthesiology and Emergency Medicine, St. Olav University Hospital, N-7006 Trondheim, Norway; 2 Norwegian University of Science and Technology (NTNU), Faculty of Medicine, Institute of Circulation and Medical Imaging, P.O. Box 8905 MTFS, N-7491 Trondheim, Norway; 3 Norwegian Air Ambulance Foundation, P.O. Box 94, N-1441 Drøbak

## Abstract

Chest compression-only (CC-only) is now incorporated in the Norwegian protocol for dispatch guided CPR (cardiopulmonary resuscitation) in cardiac arrest of presumed cardiac aetiology.

We present a case that is unique and instructive as well as unusual. It reminds us of the challenges that face bystanders, dispatch centres and ambulance services when faced with possible cardiac arrest.

This case report describes a 50 year old man in a rural community. He had suffered a heart attack 8 months previously, and was found unconscious with respiratory arrest in his garden one morning. Due to the proximity to the ambulance station, the paramedics were on the scene within three minutes. A chain-saw was lying beside him, but no external injuries were seen. The patient had no radial pulse, central cyanosis and respiratory gasps approximately every 30 seconds. Ventilation with bag and mask was given, and soon a femoral pulse could be palpated. Blood sugar was elevated and ECG (electrocardiogram) was normal. GCS (Glasgow Coma Scale) was 3. Upon arrival of the physician staffed air ambulance, further examination revealed bilateral miosis of the pupils and continuing bradypnoea. Naloxone was given with an immediate effect and the patient woke up. The patient denied intake of narcotics, but additional information from the dispatch centre revealed that he was hepatitis C positive.

After a few hours, the patient admitted to have obtained a fentanyl transdermal patch from an acquaintance, having chewed it before falling unconscious.

This case report shows the importance as well as the challenges of identifying a non-cardiac cause of possible cardiac arrest, and the value of providing causal therapy.

## Introduction

Since 2009, chest compression-only (CC-only) CPR is incorporated in the Norwegian protocol for dispatch CPR for cardiac arrest of presumed cardiac aetiology [1]. This is in accordance with recommendations from the Norwegian Resuscitation Council (http://www.nrr.org/wp-content/uploads/2009/12/NRR-om-brystkompresjoner-alene.pdf, accessed 17.06.2010). A case report illustrating the success of this approach has recently been published [2]; moreover equal efficacy of CC-only CPR compared to traditional CPR in which chest compressions are interspersed with ventilations has been shown [3], although this may not be the case in children [4]. While defaulting to CC-only CPR, the new dispatch protocol nevertheless presupposes that patients with a likely hypoxic cause of their cardiac arrest should receive standard CPR with ventilations. Drowning, strangulation and drug overdose are highlighted as reasons for suspecting hypoxia as the cause of the arrest [1]. This presupposition clearly puts a great challenge on the dispatchers to correctly identify the aetiology.

The present case report is based on interviews with the Emergency Medical System (EMS) personnel involved, the ambulance-/air ambulance case reports, and documentation from the emergency dispatch centre. The patient has given written consent to the publication. We believe it is unique and instructive as well as unusual; reminding us of the challenges that face bystanders, dispatch centres and ambulance services when faced with possible cardiac arrest.

## Case presentation

A 50 year old man living in a rural community was one morning found in his garden unconscious and in respiratory arrest. The local ambulance station was only 100 metres away, thus paramedics were on scene within three minutes. The patient was found unconscious, with Glasgow Coma Scale (GCS) 3, central cyanosis, no palpable radial pulse, and respiratory gasps approximately every 30 seconds. He was lying next to an electric chain saw, but no external injuries were seen. When applied, the AED (automated external defibrillator) showed a sinus rhythm with a frequency of 65/minute. The emergency dispatch centre was alerted, and a provisional diagnosis of cardiac arrest with pulseless electrical activity (PEA) was made. The general practitioner (GP) on call and the physician staffed air ambulance were alerted.

The dispatch central obtained and conveyed further medical information to personnel on scene: the patient suffered from insulin dependent diabetes mellitus, and had recently (8 months previously) suffered a heart attack which led to a percutaneous coronary intervention (PCI) procedure. Chest compressions were not initiated by the paramedics, however; they provided ventilation with bag and mask and oxygen. After approximately two minutes, a femoral pulse could be palpated. Blood glucose was 22 mmol/L. An ECG was recorded and transmitted via cell phone for evaluation by a cardiologist, who concluded that there was no evidence of acute cardiac pathology.

Upon arrival of the air ambulance 23 minutes after the alarm, GCS was 3, non invasive blood pressure was 140/70 mmHg, pulse 70/minute (sinus), oxygen saturation 91%, and respiratory frequency 5/minute. A Guedel tube secured the airway. Both pupils were "pin size" with no deviation. Having received additional information, the dispatch central now informed personnel on scene that the patient was positive for hepatitis C. Suspecting opioid overdose, Narcanti^® ^(naloxone hydrochloride) 0.4 mg was administered i.v. (with an additional i.m. dose), upon which the patient within a minute was awake and sat up. He was at this stage confused and could not remember what had happened. He denied chest pain, headache, nausea or breathlessness; as well as intake of any substances. Now the medical history became more complete, and details of substantial drug abuse some twenty years earlier emerged. Three hours later he admitted to having obtained a Durogesic^® ^(fentanyl) transdermal patch (75 ug/hour) from an acquaintance the previous day. He had applied it to his skin during the night, but it had fallen off. In the morning he had gone outside to do some work in the garden, had chewed it, suddenly felt very dizzy and became unconscious. The paramedics could then recall removing what they thought was chewing gum from his mouth before administering bag-mask ventilations. The patient made an unremarkable recovery.

## Discussion

This case is unusual in several ways. The alerting of the paramedics was quite unorthodox in that they were contacted directly by bystanders and not via the dispatch centre, which is the standard. Therefore, in this instance, the paramedics themselves were the first to initiate contact with the dispatch centre and define the need for further assistance.

The mode of overdose is also unusual, and we find it important to raise the awareness of the possible abuse of the transdermal patch. Literature reports show that there have been several fatalities in both adults and children following ingestion, smoking or administration via intravenous route [5,6]. The clinical pharmacokinetics of transdermal opioids are also described [7].

This case of an opioid overdose causing unconsciousness, extreme bradypnoea with hypoxia and hypotension without pulse, is in effect a case of PEA. The context could very easily lead one to wrongly infer a cardiac cause, and not an overdose. The rural setting, age of the patient and recent medical history all pointed in that direction. The electric chain saw lying by the patient could have caused further confusion as to the cause of unconsciousness; trauma and electrocution had to be ruled out. Hypoglycemia might also have been the cause of unconsciousness.

Although the dispatch centre was informed that this was a cardiac arrest, no chest compressions were given, only ventilations. The paramedics explained that the reason for this was because the patient still had respiratory gasps. We believe that differentiating between agonal respirations and severe bradypnoea is difficult, and that such a setting where no pulse could be felt should often lead to the initiation of chest compressions.

However, effective ventilations in this case reversed the hypoxia and increased cardiac output to a satisfactory level. Unconsciousness was reversed with an antidote. The question must be raised as to whether the satisfactory outcome would have been obtained if the patient had not received such prompt effective ventilations. Under usual circumstances with a longer prehospital response time, it is very likely that it would have been interpreted as an arrest of a primary cardiac cause. The protocol for dispatch guided CPR would advise compressions-only for bystanders. In an already seriously hypoxic patient, compressions-only would probably have been of little benefit to the patient.

The beauty of traditional CPR is that the combination of compressions and ventilations will cover most potentially reversible causes of cardiac arrest. Whether the simplification that follows from defaulting to CC-only really is beneficial, remains to be seen.

In conclusion, prehospital cardiac arrest poses a substantial challenge for bystanders, dispatch centres and EMS personnel. With new protocols that assume a cardiac cause by default, a high grade of awareness and suspicion is necessary. Collection and sharing of relevant clinical details, history and information is crucial.

## Competing interests

The authors declare that they have no competing interests.

## Authors' contributions

BOR drafted the manuscript. ES made substantial revisions. Both authors have revised, read and approved the article.
